# Naturally occurring human antibodies associated with AD and normal cognition

**DOI:** 10.1002/alz.089820

**Published:** 2025-01-03

**Authors:** Jorge Mauricio Reyes‐Ruiz, Jizhi Zhang, Ashutosh Kumar, Zhaoxia Yu, Charles G Glabe

**Affiliations:** ^1^ University of California, Irvine, CA USA; ^2^ University of California Irvine, Irvine, CA USA

## Abstract

**Background:**

Anti‐ Aβ monoclonal antibodies are the first FDA‐approved treatments for AD that slow cognitive decline and lower Aβ plaques. Our goal is to identify the epitope specificities of antibodies in human blood that are associated with AD and NC and determine the predicted protein targets of these antibodies.

**Method:**

101 AD (MMSE < 27) and 98 NC (MMSE > 27) serum samples were obtained from the UCI tissue repository. Epitomic profiling was conducted on the blood samples to define the epitopes for the antibodies in the samples by immunoselecting random 12 amino acid sequences from a phage display library. The resulting unique sequences are identified and their frequency counted. The 12mer sequences are tiled into tetramers and the resulting antibody‐specific tetramers are compared by Mann‐Whitney U test to identify differentially expressed epitope segments (DEES) that are upregulated in the AD and NC populations.

**Result:**

A large number of DEES (range 300 – 14,000 out of a total of 160,000 tetramers) was observed for AD and NC populations in both samples. Some of the DEES map to amyloids known to occur in AD, such as Aβ, tau, a‐synuclein, TDP43 and Tmem106b, but the actual epitopes are different in AD and NC. Both AD and NC populations’ data was then processed using UMAP for visualization and cluster analysis. Cluster analysis indicates that there are two clusters in the AD population: One large group that partially overlaps with the NC population and includes antibodies against the N‐terminus of Aβ and a well separated minor group that is defined by immunoreactivity to a heptameric linear epitope of currently unknown origin. The NC population includes several groups that contain anti amyloid antibodies.

**Conclusion:**

Human blood contains a large number of antibodies that are differently expressed in AD that recognize distinct amyloid and non‐amyloid epitopes. These antibodies may be protective for disease, predictive for presymptomatic disease onset and progression or antibodies in response to amyloid deposition that could be protective depending on the concentration or predictive of disease subtype. Further work is aimed at determining the significance of the different antibodies and validating their disease association

